# Mapping the scientific research on integrated care: a bibliometric and social network analysis

**DOI:** 10.3389/fpsyg.2023.1095616

**Published:** 2023-09-14

**Authors:** Dandan Guo, Chaofeng Zhou, Haomiao Li, Dai Su, Guangwen Gong, Xinlin Chen, Xinlan Chen, Yingchun Chen

**Affiliations:** ^1^School of Medicine and Health Management, Tongji Medical College, Huazhong University of Science and Technology, Wuhan, China; ^2^Wuhan Library, Chinese Academy of Sciences, Wuhan, China; ^3^Department of Library, Information and Archives Management, School of Economic and Management, UCAS, Beijing, China; ^4^School of Political Science and Public Administration, Wuhan University, Wuhan, China; ^5^Department of Health Management and Policy, School of Public Health, Capital Medical University, Beijing, China; ^6^Guangwen Gong, School of Management, Hubei University of Chinese Medicine, Wuhan, China; ^7^Union Hospital, Tongji Medical College, Huazhong University of Science and Technology, Wuhan, China; ^8^Tongji Hospital, Tongji Medical College, Huazhong University of Science and Technology, Wuhan, China; ^9^Research Centre for Rural Health Service, Key Research Institute of Humanities and Social Sciences of Hubei Provincial Department of Education, Wuhan, China

**Keywords:** integrated care, bibliometric, social network analysis, Citespace, Web of Science

## Abstract

**Background:**

Integrated care (IC) is the cornerstone of the sustainable development of the medical and health system. A thorough examination of the existing scientific literature on IC is essential for assessing the present state of knowledge on this subject. This review seeks to offer an overview of evidence-based knowledge, pinpoint existing knowledge gaps related to IC, and identify areas requiring further research.

**Methods:**

Data were retrieved from the Web of Science Core Collection, from 2010 to 2020. Bibliometrics and social network analysis were used to explore and map the knowledge structure, research hotspots, development status, academic groups and future development trends of IC.

**Results:**

A total of 7,501 articles were obtained. The number of publications on IC was rising in general. Healthcare science services were the most common topics. The United States contributed the highest number of articles. The level of collaboration between countries and between authors was found to be relatively low. The keywords were stratified into four clusters: IC, depression, integrative medicine, and primary health care. In recent years, complementary medicine has become a hotspot and will continue to be a focus.

**Conclusion:**

The study provides a comprehensive analysis of global research hotspots and trends in IC, and highlights the characteristics, challenges, and potential solutions of IC. To address resource fragmentation, collaboration difficulties, insufficient financial incentives, and poor information sharing, international collaboration needs to be strengthened to promote value co-creation and model innovation in IC. The contribution of this study lies in enhancing people’s understanding of the current state of IC research, guiding scholars to discover new research perspectives, and providing valuable references for researchers and policymakers in designing and implementing effective IC strategies.

## 1. Introduction

With the changes in disease spectrum, the influence of social, natural environment and lifestyle on health became increasingly prominent. However, the fragmented healthcare service system and the single clinical discipline treatment method cannot effectively respond to patient requirements. In response to the challenges being faced by health systems, the World Health Organization (WHO) proposed the Integration of Health Care Delivery (IHCD) in 1996 (Integration of Health Care Delivery, 1996), and People-Centered Integrated Care (PCIC) strategy in 2015 (World Health Organization, 2015). These initiatives reflect the growing recognition of the importance of IC in improving health outcomes. However, the literature suggests that there is no single definition of IC, defining IC is an ongoing process, and its definition continues to evolve as the health system and environment change (Kodner and Spreeuwenberg, 2002; Lennox-Chhugani, 2021). Despite the absence of a clear definition, scholars have reached some consensus on the fundamental principles and goals of IC, and countries worldwide have been exploring tailored IC practices that suit their national contexts, which have demonstrated improvements in quality, efficiency, accessibility, and cost management of healthcare systems (Sweeney et al., 2007; Dudley and Garner, 2011; Maruthappu et al., 2015; Coates et al., 2022). Moreover, numerous studies have shown that achieving IC can generate significant health benefits, especially for the older populations, those with chronic conditions, the mentally ill, and other special people who need long-term care, such as reducing anxiety and fatigue in cancer patients, improving quality-adjusted life years in pediatric patients with asthma, and improving health-related quality of life while decreasing behavioral problems, as well as reducing caregiver burden in dementia patients (Lengacher et al., 2009; Andersen, 2014; Sun et al., 2019; Duenas-Meza et al., 2020; Schad et al., 2020; Ha et al., 2021). Some studies also showed that patients who received a fully integrated primary-secondary care model were more satisfied than patients who received treatment separately from the primary or secondary care sectors, and such patients could achieve better health outcomes (Uga et al., 2017; van Olmen et al., 2020; Donald et al., 2021). In addition, IC expands the perspective of clinical care from the biomedical to the biopsychosocial by treating comorbid psychiatric illness; IC also has significant effects on controlling symptoms and improving the physical fitness of schizophrenic patients (Smith, 2009; Sharpe et al., 2020). IC has become increasingly important in health care policy, multidisciplinary collaboration and clinical practice as a means to achieve high-quality services (Lewis et al., 2018).

From an academic perspective, extensive systematic reviews on IC have been undertaken with various goals in the past 10 years. For example, Hughes et al. (2020) identified an array of strategies and conceptual work of IC. Zonneveld et al., (2018) developed a set of underlying values of IC and discussed the practical applications and their uses. Rocks et al. (2020) evaluated the economic benefits of IC and suggested that IC is likely to reduce costs and improve outcomes. Bautista et al. (2016) developed a comprehensive framework to provide evidence on the state of the art in measuring IC. Overall, many studies have been undertaken on IC, but these analyses have mainly focused on specific aspects of IC, and comprehensive systematic analyses have been limited. Bibliometrics could quantitatively analyze scientific publications, but few bibliometric studies have been performed on research relating to IC. In 2013, Sun used the Bibliographic Item Co-occurrence Matrix Builder and SPSS to analyze the growth pattern, jurisdiction distribution, core journals and key research domains of IC (Sun et al., 2014). Li retrieved articles from 1997 to 2016, used Histcite and VOS viewer to analyze publication numbers and citations, and co-authorship between countries and institutions and clusters of IC (Li et al., 2020). However, these two studies lacked the most recent data, single analysis tool was used and the data could not be fully mined. Therefore, we use a variety of tools to comprehensively analyze the status of IC through bibliometric analysis and social network analysis (SNA) to reveal the development status in multiple dimensions of IC to date. Specifically, we use publication growth trend, disciplinary areas distribution, international productivity and collaboration, author productivity and collaboration, citation analysis and keyword occurrence research hotspots and trends.

## 2. Materials and methods

### 2.1. Data sources

Data illustrated here were retrieved from the Web of Science Core Collection (WOSCC), which is the most frequently used citation database for bibliometric analysis, and the structured data format provides considerable convenience to quantitative analysis. This study selects articles from four databases, namely, Science Citation Index Expanded, Social Sciences Citation Index, Conference Proceedings Citation Index-Science and Conference Proceedings Citation Index—Social Science & Humanities, as the data source.

### 2.2. Search strategy

Advanced search of WOSCC is used, and the research strategy was as follows: {[TS = (“integrat* care” OR “integrat* health” OR “integrat* healthcare” OR “care integration” OR “integrat* of care” OR “integrat* medic*”)]}. The publication time span was limited to 2010.10.1–2020.10.12, and 7,501 publications were collected. Document Type: Article.

### 2.3. Analysis methods

#### 2.3.1. Bibliometrics analysis method

Bibliometrics is one of the key methods to objectively measure the influence of academic publications (Agarwal et al., 2016). It uses methods such as mathematical modeling, statistical analysis and SNA to explore the knowledge structure, research hotspots, development status, academic groups and future development trends of a certain field (Zhu and Guan, 2013).

CiteSpace and VOSviewer visualization software were used to depict the national cooperative science knowledge mapping. Nodes represent the author, institution, country or cited reference. The links between nodes represent the collaboration or co-occurrence relationships. The thickness of the connection represents the strength of cooperation. The purple node indicates high BC and acts as pivotal points in a field (Chen, 2006). In addition, we used Price’s law, which is an indicator for analyzing productivity in a specific field (Lópezmuñoz et al., 2013). The lower limit for the number of publications in the core author group or the lower limit of the frequency of high-frequency keywords (*M*) is $M=0.749\sqrt{N_{\text{max}}\;}$. N_max_ represents the maximum value of the number of posts or the maximum value of frequency.

#### 2.3.2. SNA

SNA is a method of studying nodes and their relationships. SNA can effectively identify the influence of each node and the interaction between nodes in the social network. The indicators to measure the influence of nodes are betweenness centrality (BC), closeness centrality (CC), and degree centrality (DC). BC represents the node’s ability to control the connection between two non-adjacent nodes. The larger the value, the stronger the control ability of the entire network information flow, and is at the core of the entire network (Chen, 2005). CC represents the distance between the nodes. The smaller the distance, the larger the value, indicating that the node is in an important position in the entire network. DC is the number of nodes directly connected to the node. The larger the value represents the higher the importance, but it does not mean that the node is in the center of the network (Tonta and Darvish, 2010). Moreover, Ucinet and NetDraw were used for SNA in this study. Ucinet is a software package for social network analysis that provides tools for data management, analysis (Borgatti et al., 2002), and visualization. It can be used to analyze social network data and identify patterns in the structure of relationships between individuals or organizations. NetDraw is a visualization tool that can be used in conjunction with Ucinet to create network diagrams and visualize social network data (Borgatti, 2002; Cronin, 2015).

### 2.4. Tools

**Figure 1:**
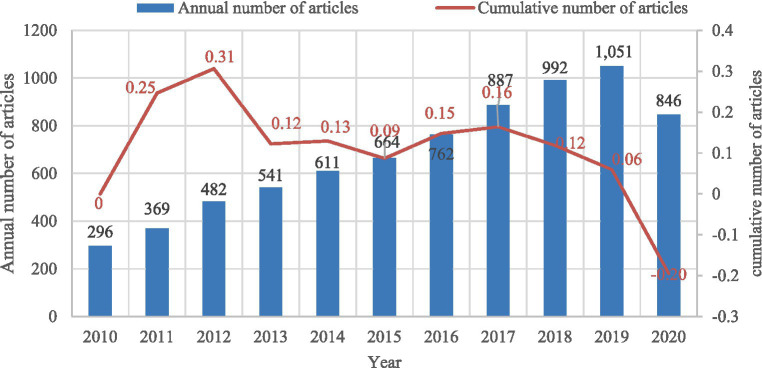
Annual article counts on IC from 2010 to 2020.

**Figure 2:**
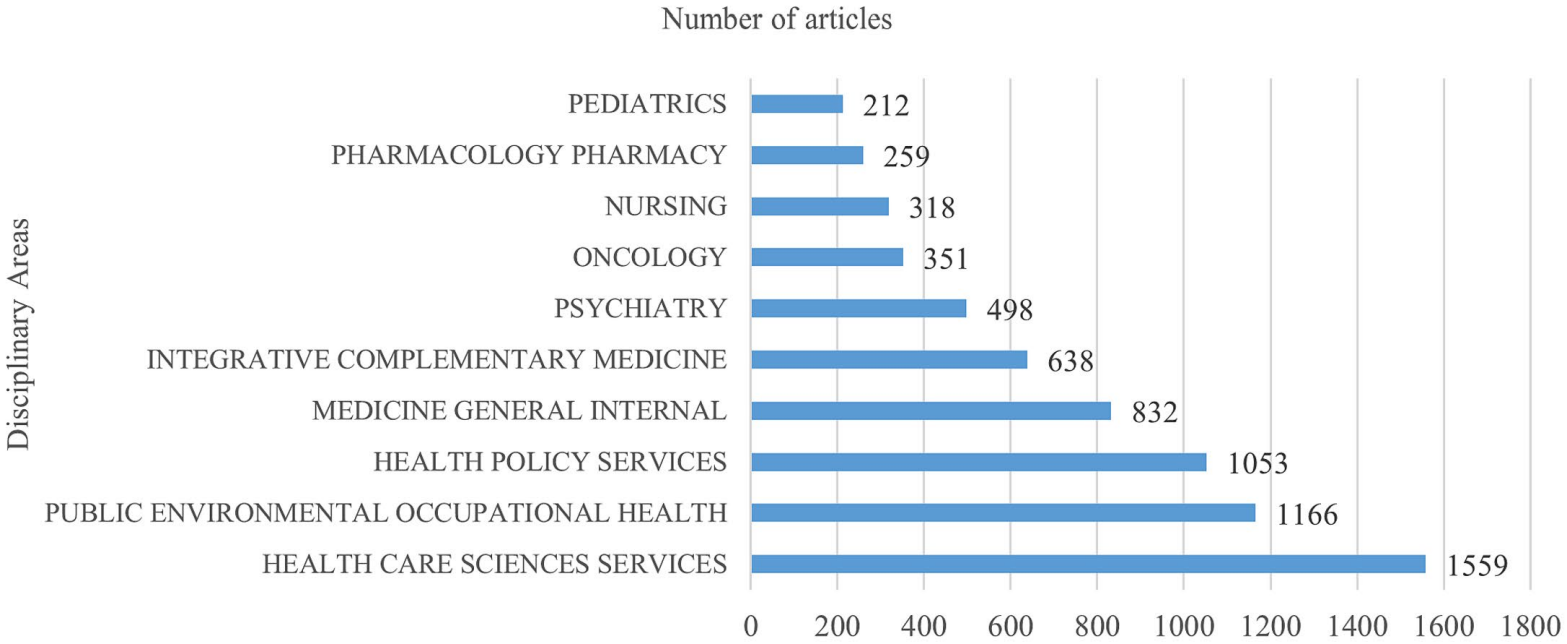
Distribution of disciplinary areas on IC.

**Figure 3:**
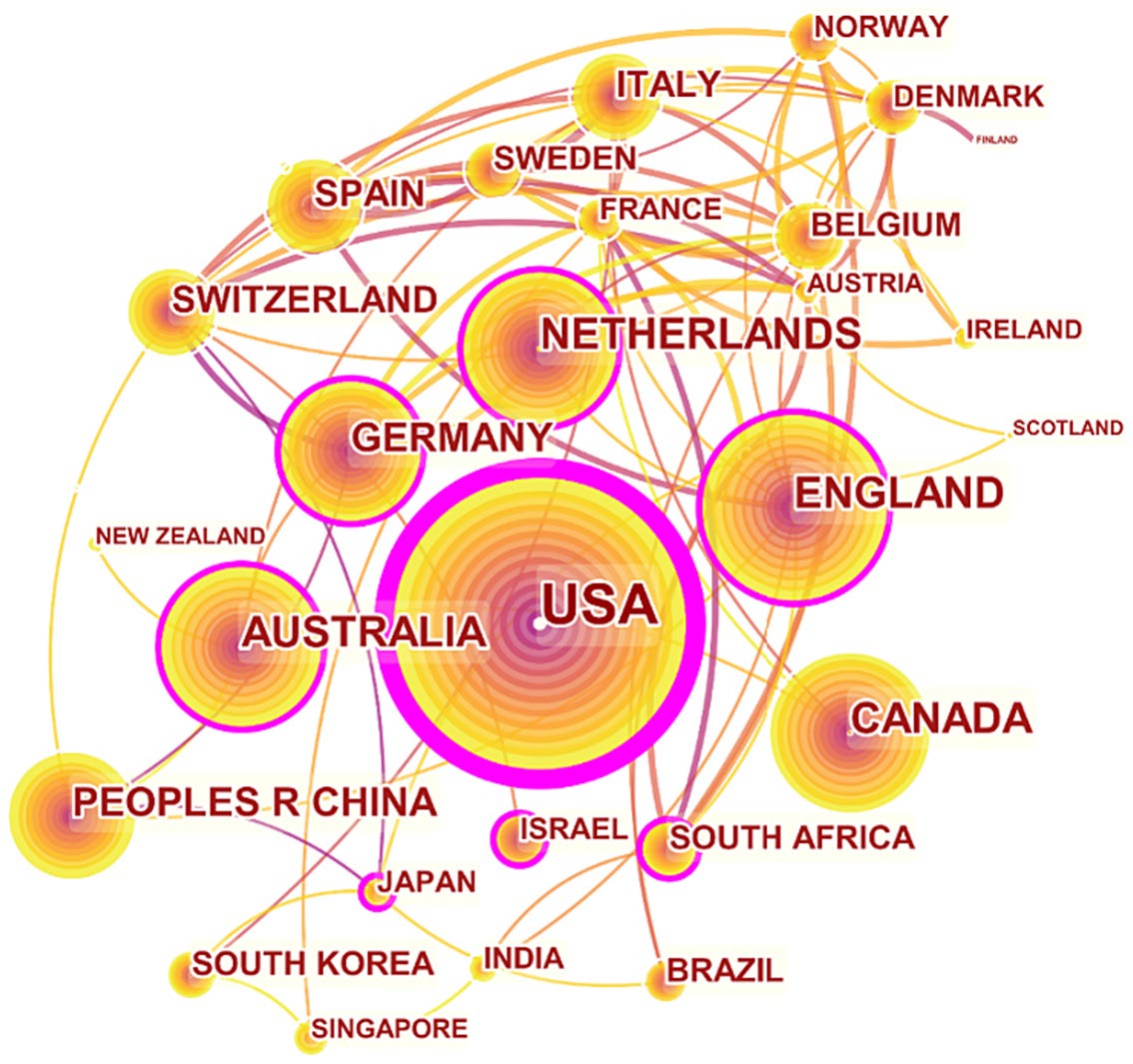
International collaboration network of the 20 most productive countries.

**Figure 4:**
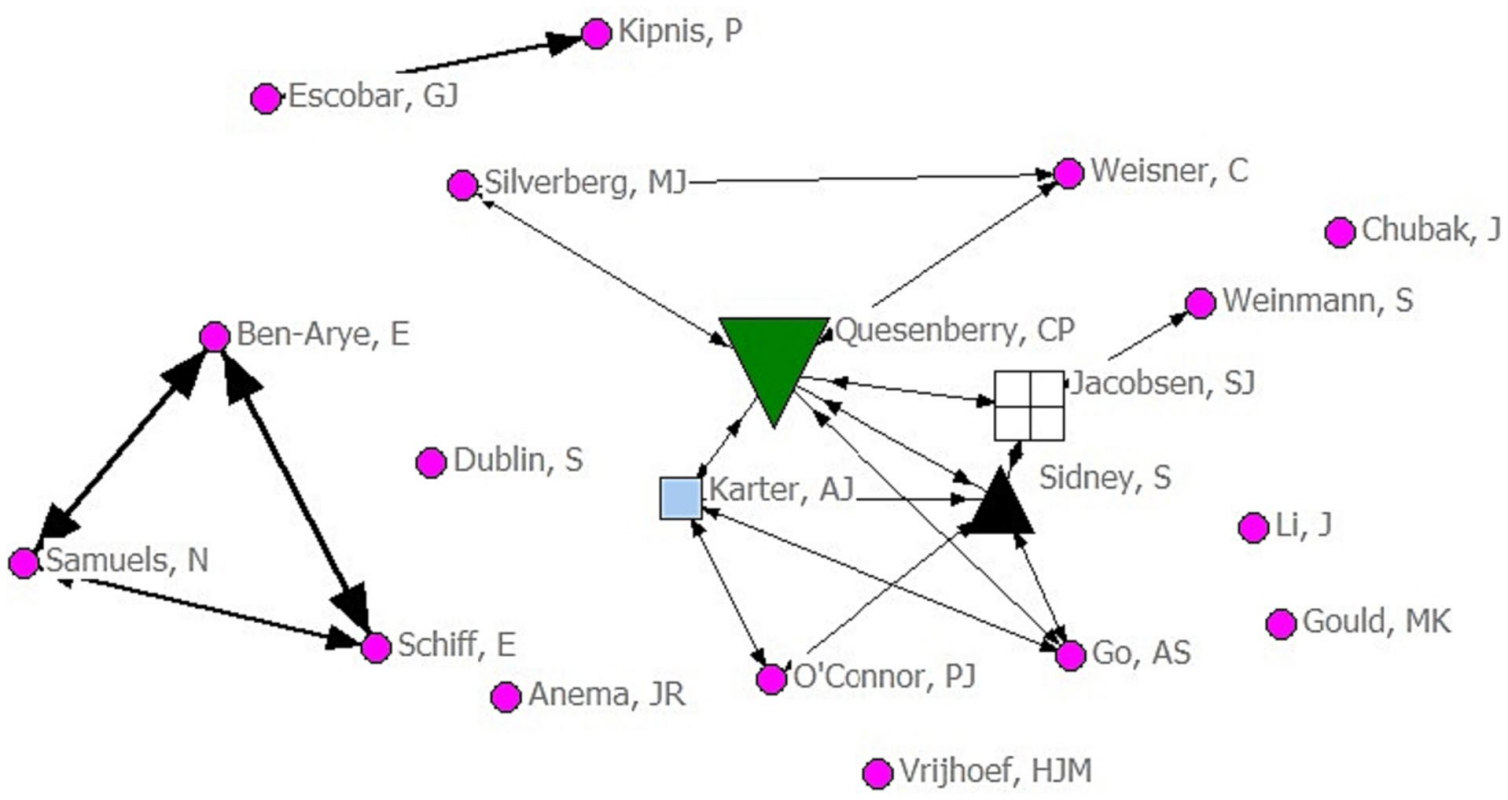
Collaboration network of top 20 most productive authors.

**Figure 5:**
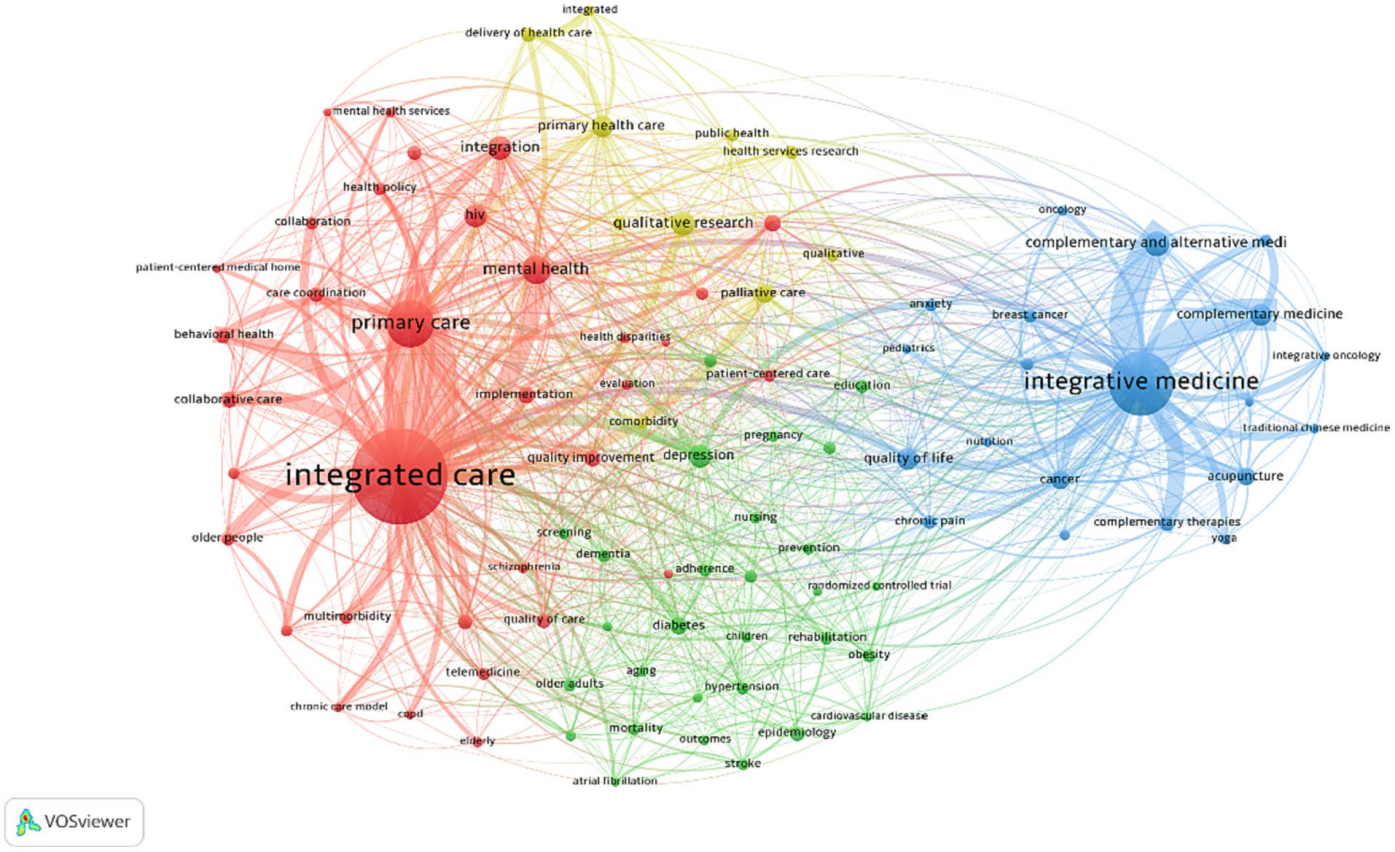
Keyword co-occurrence network of the most frequent keywords by the counts.

**Figure 6:**
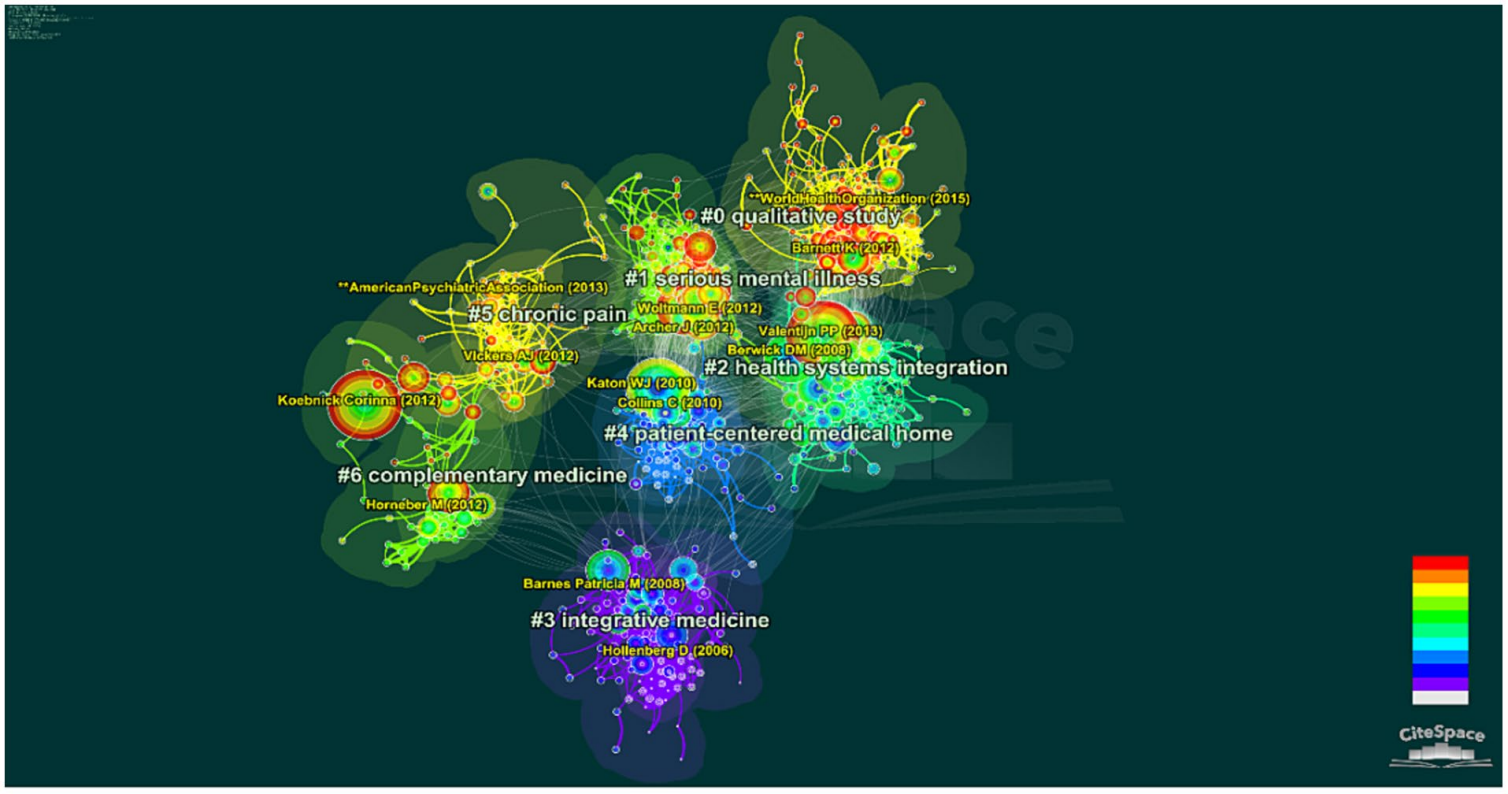
Document co-citation network.

**Figure 7:**
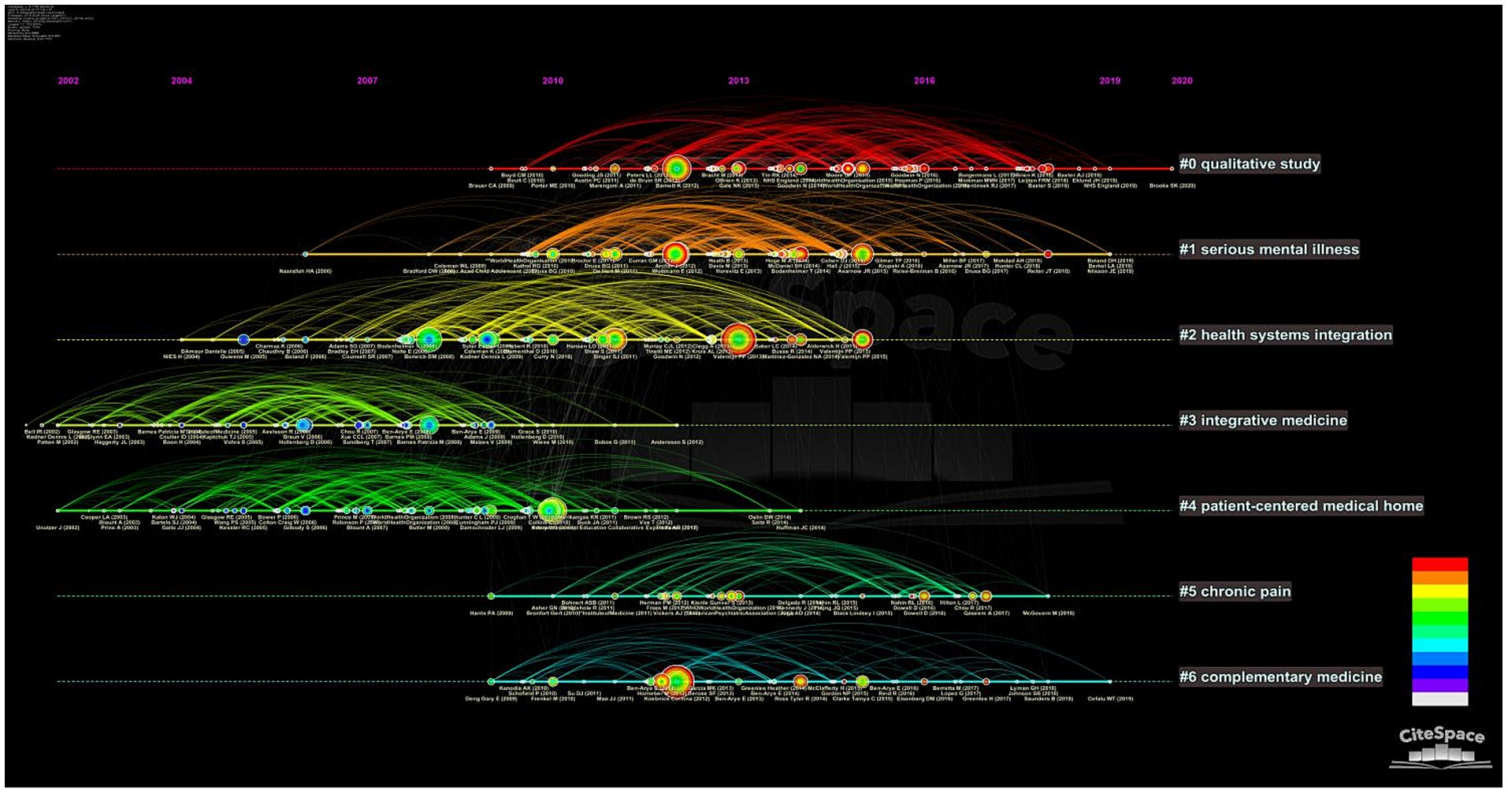
Document co-citation analysis clusters timeline visualization.

**Figure 8:**
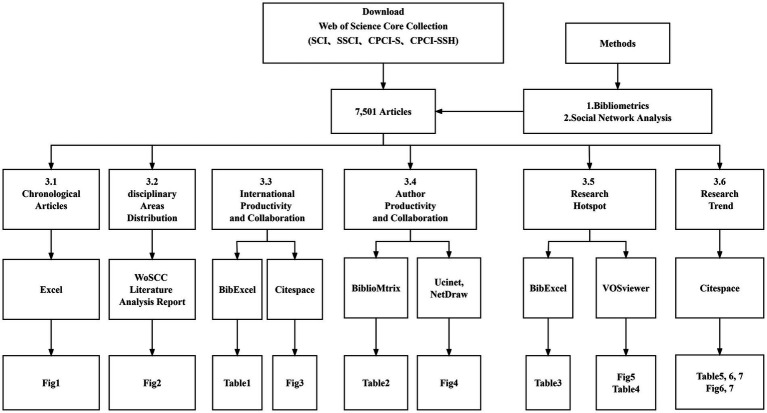
Flowchart of this paper.

Firstly, we use Excel’s bar graph to count the annual number of posts and the rate of change of posts (Figure 1). Secondly, we used the WOSCC Literature Analysis Report to find out the distribution of disciplinary areas (Figure 2). We then used BibExcel to extract the top 20 high-yield countries (Table 1; Persson et al., 2009). We used the CiteSpace visualization tool to depict the mapping of international productivity and collaboration (Figure 3; Chen, 2006). Next, we use R Voice’s BiblioMetrix to extract the number of articles posted by highly productive authors and other indexes (Table 2; Aria and Cuccurullo, 2017). Then, we used the Bibliographic Item Co-Occurrence Matrix Builder to extract the author co-occurrence matrix and imported SNA software Ucinet and NetDraw for drawing (Figure 4; Liu, 2009). We then used BibExcel to extract high-frequency keywords (Table 3) and used VOSviewer visualization software (Figure 5) to draw keyword co-occurrence knowledge mapping (keyword co-occurrence knowledge mapping) and the key in each word cluster (Table 4; van Eck and Waltman, 2010). Finally, we used CiteSpace to draw reference co-citation knowledge mapping (Table 5; Figure 6), highly cited documents (Table 6) and timelines (Figure 7). Specific tools and diagrams are shown in Figure 8.

**Table 1:** Top 20 countries in terms of number of articles and BC.

Country	Territory	Frequency	BC	Country	Territory	Frequency	BC
USA	North America	4,113	0.45	Sweden	Europe	136	0.06
England	Europe	656	0.16	Belgium	Europe	12	0.06
Australia	Oceania	501	0.15	South Africa	Africa	14	0.12
Netherlands	Europe	475	0.15	South Korea	Asia	7	0.04
Canada	North America	469	0.05	Brazil	Latin America	9	0.05
Germany	Europe	356	0.11	Denmark	Europe	13	0.05
Peoples R China	Asia	312	0.08	Israel	Asia	11	0.12
Spain	Europe	189	0.02	France	Europe	14	0.06
Italy	Europe	186	0.08	Norway	Europe	9	0.01
Switzerland	Europe	177	0.07	India	Asia	8	0.04

**Table 2:** Impact of top 20 most productive authors ranked by papers.

Ranking	Authors	# of papers	BC	CC	DC	h-index	g-index
1	Ben-Arye E	60	0	342	2	13	19
2	Schiff E	43	0	342	2	12	17
3	Jacobsen SJ	33	7	233	3	15	29
4	Escobar GJ	32	0	361	1	14	27
5	Go AS	32	0	234	3	16	32
6	Karter AJ	31	2	233	4	14	31
7	Quesenberry CP	31	14	230	6	13	31
8	Samuels N	31	0	342	2	8	12
9	Li J	26	0	380	0	10	19
10	O’Connor PJ	25	0	237	0	14	25
11	Sidney S	25	6	231	5	16	25
12	Vrijhoef HJM	25	0	380	0	7	15
13	Chubak J	24	0	380	0	13	21
14	Kipnis P	24	0	361	0	14	24
15	Silverberg MJ	24	0	236	2	10	20
16	Anema JR	22	0	380	0	10	22
17	Gould MK	22	0	380	0	8	17
18	Weinmann S	22	0	240	1	10	22
19	Weisner C	22	0	236	2	12	22
20	Dublin S	21	0	380	0	12	21

**Table 3:** Most highly frequent keywords ranked by counts.

Ranking	Keyword	Counts	Ranking	Keyword	Counts	Ranking	Keyword	Counts
1	Integrated care	1,005	33	Health policy	48	64	cost-effectiveness	28
2	Integrative medicine	547	34	Health services	48	65	Mixed methods	28
3	Primary care	349	35	Public health	48	66	Aged	27
4	Mental health	167	36	Qualitative	47	67	CAM	27
5	Complementary and alternative medicine	137	37	Alternative medicine	46	78	General practice	27
6	Qualitative research	131	38	Pregnancy	46	69	Health information technology	27
7	Integration	129	39	Prevention	44	70	Medicaid	27
8	Complementary medicine	115	40	Nursing	43	71	Medication adherence	27
9	Primary health care	115	41	Mortality	42	72	Patient experience	27
10	Quality of life	110	42	Serious mental illness	41	73	Qualitative study	27
11	Palliative care	90	43	Evaluation	40	74	Asthma	26
13	Quality improvement	73	44	Integrated	40	75	Case management	26
14	Collaborative care	69	45	Self-management	40	76	Patient safety	26
15	Integrated health care	64	46	Medical education	39	77	Physical activity	26
16	Quality of care	64	47	Aging	36	78	Risk factors	26
17	Chronic Disease	63	48	Elderly	36	79	Spirituality	26
18	Complementary therapies	63	49	Health disparities	36	80	Telehealth	26
19	Behavioral health	61	50	Mental health services	36	81	Chiropractic	25
20	Care coordination	61	51	Chronic care model	35	82	Health systems	25
21	Delivery of Health care	59	52	Diabetes mellitus	35	83	Herbal medicine	25
22	Chronic pain	58	53	Health	32	84	Mental health care	25
23	Health services research	55	54	Supportive care	32	85	Pain management	25
24	Older adults	52	55	Traditional Chinese medicine	32	86	Assessment	24
25	Patient-centered care	52	56	COPD	31	87	Chronic care	24
26	Collaboration	51	57	Healthcare	31	88	Disability	24
27	Health care	51	58	Health care reform	30	89	Implementation science	24
28	Telemedicine	51	59	Patient-centered medical home	30	90	Integrated healthcare	24
29	Health promotion	50	60	Chinese medicine	29	91	Interprofessional education	24
30	Older people	50	61	Integrated health care systems	29	92	Long-term care	24
31	Rehabilitation	49	62	Intervention	29	93	Prevalence	24
32	Electronic health records	48	63	Chemotherapy	28	94	Program evaluation	24

Count, Frequency in 7,501.

**Table 4:** Keywords for the four clusters.

Cluster	Cluster name	Author keywords
1(Red,36)	Integrated care	assessment, behavior health, care coordination, chronic care, chronic care model, chronic disease, collaboration, collaborative care, copd, elderly, evaluation, general practice, health care, health disparities, health systems, implementation science, integrated care, integrated health care, integration, interpersonal education, intervention, long-term care, Medicaid, mental health, mental health care, mixed methods, multimorbidity, older people, patient experience, patient-centered home, primary care, qualitative, quality study, quality improvement, quality of care, serious mental illness
2(Green, 26)	Depression	asthma, aging, aged, case management, cost-effective, diabetes mellitus, disability, electronic health record, health, health information technology, health promotion, health services, healthcare, heart failure, integrated healthcare, medication adherence, nursing, older adults, patient-centered care, prevalence, prevention, rehabilitation, risk factors, self-management, telehealth, telemedicine
3(Blue,19)	Integrative medicine	alternative medicine, cam, Chinese medicine, chronic pain, complementary and alternative medicine, complementary medicine, complementary therapies, herbal Medicine, integrative medicine, integrative oncology, medical education, pain management, physical activity, quality of life, spirituality, supportive care, traditional Chinese medicine, adherence, chemotherapy
4(Yellow,12)	Primary health care	delivery of health care, health care reform, health policy, health services research, integrated, integrated health services, mental health services, palliative care, primary health care, program evaluation, public health, qualitative research

**Table 5:** Top seven largest clusters ranked by size.

Cluster#	Label (LLR)	Size	Silhouette	Top 3 terms (LLR)
0	Qualitative study	129	0.862	Integrated patient care (2.64), requiring medical care (2.25), noncommunicable diseases (2.25).
1	Serious mental illness	122	0.890	Mental health service use (1.43), mental health program (1.32), severe psychotic disorder (1.26).
2	Health systems integration	119	0.845	Therapeutic assertive community treatment (2.98), comprehensive integrated control program (2.77), stratification strategies (2.77).
3	Integrative medicine	104	0.928	Integrated goal-directed approach (0.67), western model (0.4), oncology clinic (0.4).
4	Patient-centered medical home	94	0.869	Patient outcomes research (0.67), providing ward pharmacy service (0.61), patient adherence (0.58).
5	Chronic pain	55	0.921	Physical chronic health care need (0.47), chronic somatic diseases (0.43), chronic pain result (0.43).
6	Complementary medicine	50	0.920	Teaching yoga (0.72), traditional Chinese medicine (0.66), multidisciplinary care program (0.62).

**Table 6:** Top 10 most frequently cited articles during 2010–2020 sorted by count.

Author (year)	Journal	Count	BC	Cluster#
Valentijn et al. (2013)	International Journal of Integrated Care	117	0.13	2
Kodner and Spreeuwenberg (2002)	International Journal of Integrated Care	90	0	6
Berwick et al. (2008)	Health Affair	68	0.08	2
Barnett et al. (2012)	Lancet	65	0.05	0
Woltmann et al. (2012)	American Journal of Psychiatry	57	0.16	1
Katon (2010)	New England Journal Medical	55	0.37	4
Singer et al. (2011)	Medical Care Research Review	53	0.13	0
Archer (2012)	Cochrane Database of Systematic Reviews	52	0.06	1
Barnes et al. (2008)	National Health Statistics Reports	45	0.04	3
Horneber et al. (2012)	Integrative cancer therapies	43	0.01	6

## 3. Results

### 3.1. Chronological articles

Figure 1 presents the number of related papers published from 2010 to 2020. The number of publications on IC was rising in general. However, the annual growth rate of related literature varies greatly in this field; the highest literature volume is five times larger than the lowest (2012 and 2019).

### 3.2. Disciplinary areas distribution

Figure 2 shows the distribution of the disciplinary areas on IC. The total number of documents in the top 10 disciplines is 6,886, accounting for 91.107%, namely, health care science services (1,559, 20.627%), public environmental occupational health (1,166, 15.427%), health policy services (1,053, 13.932%), general and internal medicine (832, 11.008%), integrative complementary medicine (638, 8.441%), psychiatry (498, 6.589%), oncology (351, 4.644%), nursing (318, 4.207%), pharmacology pharmacy (259, 3.427%) and pediatrics (212, 2.805%).

### 3.3. International productivity and collaboration analysis

Table 1 shows the top 20 countries ranked by the percentage of publications. The United States was the most productive country, with a total of 4,112, accounting for 54.82%, and the BC is 0.45, followed by the United Kingdom (656, 8.75%, 0.16) and Australia (501, 6.68%, 0.15). In terms of regions, Europe has 11 countries, accounting for 55%; Asia has 4 (20%), America has 3 (15%), and Oceania and Africa have 1 each (5% respectively). Combining Table 1 and Figure 3, the United States, with the largest number of publications and the highest BC, is at the center of the national cooperation network. In addition, countries such as the United Kingdom, Australia and the Netherlands have close cooperation with other countries, and they play important roles in the development of global IC. China has published 312 articles (4.16%) and ranks first in Asia, but the BC is only 0.08. showing that China lacks international cooperation with other countries on IC.

### 3.4. Author productivity and collaboration

The BiblioMetrix Tool Package in R development software was used to analyze all the authors in 7,501 studies, with a total of 31,958 authors. The number of authors per paper was 4.26, and the number of authors with one paper was 25,411, accounting for 79.5%.

According to Price’s Law, authors of more than or equal to six articles (the core author), a total of 598 authors, and only the top 20 prolific authors are selected in this study. Then, software to calculate multiple indicators such as the number of essays, BC, CC, DC, h-index and g-index for the top 20 authors; multi-dimensional judgment of the author’s influences is developed (Hirsch, 2005; Egge, 2006). As shown in Table 2, Ben-Arye E has published the most articles, with a total of 60 articles, followed by Schiff E and Jacobsen SJ.

Figure 4 shows the collaborative network of the top 20 productive authors. The nodes represent 20 core authors; The circle means BC is 0, the diamond means BC is 2, the upward-pointing triangle means BC is 6, the box means BC is 7, and the downward-pointing triangle means that BC is 14. Combining Table 2 and Figure 4, although Quesenberry CP does not have the largest number of articles, the BC is the largest, so he is at the center of the network. In addition, Quesenberry CP, Jacobsen SJ, Sidney S, and Karter AJ formed an academic group, who are from the same institution (Kaiser Permanente, Oakland, California, United States). The other academic group was formed by Ben-Arye E, Schiff E, and Samuels N. However, no cooperation occurred between the two groups. In addition, six authors did not cooperate with other authors.

### 3.5. Research hotspots

Noun items can be used to detect research hotspots in the field (Chiu and Ho, 2007). Keywords are nouns or phrases that reflect the core content of a publication (Chen et al., 2018), and high-frequency words represent the research hotspots (Xie et al., 2008). BibExcel was used to extract all the keywords in the literature, and after merging and removing duplication, a total of 12,449 keywords were obtained. Among them, 9,347 keywords appeared once, accounting for 75.08%. According to Price’s Law, keywords with a frequency greater than or equal to 24 are considered high-frequency keywords. The top ten keywords by frequency are “IC” (1005), “integrative medicine” (547), “primary care” (349), “mental health” (167), “complementary and alternative medicine” (137), “qualitative research” (131), “integration” (129), “complementary medicine” (115), “primary health care” (115), and “quality of life” (110), as shown in Table 3.

VOSviewer was used to draw map keyword co-occurrence networks, as shown in Figure 5.

A total of 4 clusters and 1,144 lines were formed based on the Linlog/Modularity algorithm. These 4 clusters represent the research hotspots on IC in the past decade. Using the largest node in each research hotspot as the cluster name, Cluster 1 (Red, IC) is the largest category and contains 36 keywords. Cluster 2 (Green, Depression) contains 26, Cluster 3 (Blue, Integrative medicine) contains 19, and Cluster 4 (Yellow, Primary health care) contains 12, as shown in Table 4.

The keywords in the four clusters reveal the characteristics involved in the development of integrated healthcare. ① IC should be value-based and health-centered, providing health services across the life cycle and achieving optimal health outcomes with limited resources. ② Service targets should include the whole population, not just special people who need long-term care.③ Focus more on health promotion and disease prevention, and to promote the integration of public health services and clinical care. ④ Pay attention to self-management and be the first responsible person for own health. ⑤ Attach importance to the role of complementary medicine in IC. ⑥ IC strategies need to be designed and developed taking account of the particular local realities.⑦ Promote incentive reform and give full play to the regulatory role of health insurance leverage to promote the development of IC. ⑧ Use digital technology to empower IC. ⑨ Establish effective governance, evaluation and accountability mechanisms.

### 3.6. Research trend

#### 3.6.1. Reference co-citation analysis

Document co-citation analysis is often used to detect historical evolution and research trends in the field (Chen et al., 2012). A total of 210,081 references are attached to 7,501 articles. CiteSpace was used to analyze these references. A total of 850 nodes and 3,176 lines were mapped. To highlight the role of key documents, the top 50 cited documents per year were selected as the analysis object.

Based on the log-likelihood ratio algorithm (Fang et al., 2018), and according to the silhouette value (the closer the value is to 1, the more reliable the clustering), clusters less than 0.7 were filtered out, finally forming seven clusters. The top three clusters were Cluster 0 (qualitative study), Cluster 1 (serious mental illness), and Cluster 2 (health systems integration), as shown in Figure 6 and listed in Table 5.

#### 3.6.2. Highly cited articles analysis

Cited references have made revolutionary contributions to the entire research field. Therefore, co-citation analysis of highly cited references is helpful to detect the research foundation in the field (Hou et al., 2018). The largest node in each cluster in Figure 6 can be observed, and Table 6 lists the top 10 cited references according to their citation frequency and their categories.

As shown in Table 7, there is little stable understanding of IC meant, but all emphasize that IC guiding principle is person-centered, the core purpose is to improve the efficiency of the health system and ensures people receive a continuum health services. Moreover, coordination among stakeholders is critical to the accomplishment of goals. In general, the forms of IC are complex and multidimensional. The scope of integration includes organizational, services, funding, information, and regulatory. In terms of service recipients, the disadvantaged are the ones who get the most attention, IC can generate significant health benefits for them.

**Table 7:** Viewpoints of the top 10 most frequently cited articles.

Author	Title	Viewpoint	Dimensions of IC	Core elements
Valentijn PP	Understanding integrated care: a comprehensive conceptual framework based on the integrative functions of primary care	Integration has to be pursued at different levels within a system based on specific contexts to provide continuous, comprehensive and coordinated service to individuals and populations.	Micro (clinical); meso (professional-and organizational); macro (system); functional (nancial, management and information systems); normative (shared mission, vision, values and culture)	Person-focused, comprehensive care, inter-sectorial partnerships, coordinate and support accountability and decision-making, common frame
Kodner DL	Integrated care: meaning, logic, applications, and implications--a discussion paper	There are varying degrees of completeness, comprehensiveness and formality in IC, the use of strategies would depend on the characteristics of the patient population and the specific challenges.	Funding, administrative, organization, service, clinical	Patient-oriented, connectivity, alignment and collaboration, cost-effectiveness.
Berwick DM	The Triple Aim: Care, Health, And Cost	Specifying a population of concern, policy constraints, integrator are prerequisites for improving the individual experience of care, improving the health of populations, and reducing the *per capita* costs of care for populations.	Macro system, financial, organization, service, clinical	Patients need, shared plans, coordinating care, efficient and equitable, reduction and control costs.
Barnett K	Epidemiology of multimorbidity and implications for health care, research, and medical education: a cross-sectional study	For most multimorbid patients, a generalist clinicians to provide personalized, comprehensive continuity of care is needed, especially in socio-economically deprived areas.	Service, clinical	Personalized, comprehensive
Woltmann E	Comparative effectiveness of collaborative chronic care models for mental health conditions across primary, specialty, and behavioral health care settings: systematic review and meta-analysis	Collaborative chronic care models can improve outcomes for individuals with mental disorders.	Service, clinical, information	Collaborative, combined modality therapy, continuity care, reduction and control costs.
Katon WJ	Collaborative Care for Patients with Depression and Chronic Illnesses	Coordinated care management of multiple conditions improves medical outcomes and depression for patients with depression and chronic illnesses.	Clinical	Coordinate, individualized treatment, treat-to-target
Singer SJ	Defining and measuring integrated patient care: promoting the next frontier in health care delivery	The challenge in delivering integrated is to provide optimal amounts of both coordination and patient centeredness.	system, organization, Service, clinical	Coordination, continuous, Patient centeredness
Archer J	Collaborative care for depression and anxiety problems.	Collaborative care is associated with significant improvement in depression and anxiety outcomes compared with usual care.	Service, clinical	Collaborative, reduce burden
Barnes PM	Complementary and alternative medicine use among adults and children: United States, 2007	CAM purports to prevent or treat disease, the use of CMA by children is significantly lower than adults, and adults mainly use CAM for pain relief.	Service, clinical	Prevent illness, relief pain
Horneber M	How Many Cancer Patients Use Complementary and Alternative Medicine	Patients use CMA as a coping strategy to treat disease, on average, half of cancer patients use CAM, and it needs for clear strategies to deal with this prevalent health-related behavior.	Clinical	Frequency and patterns

#### 3.6.3. Cluster timeline visualization

To further clarify the historical evolution of research hotspots in the IC field, the timeline visualization was mapped based on 210,081 references. In Figure 7, references are shown in the form of circles whose thickness indicates the number of citations within the time slice. The citation tree-ring color represents the citation time, and red indicates that the cited reference has a high BC value. Figure 7 shows the internal and inter-relationship of the largest 7 clusters. The first cluster to emerge was Cluster3 (integrative medicine), which outlined the relevance of complex systems theory as an approach to health outcomes research in the context of integrative medicine. This theory recognizes that health outcomes are influenced by various complex and interconnected factors, including biological, psychological, social, and environmental factors. Cluster4 (patient-centered medical home), which focused on depressed older adults in primary care settings. However, these two research hotspots have not gained much attention in recent years. Although Cluster0 (qualitative study) appeared later, it gathered many red nodes from 2010 to 2018, implying that many highly cited references emerged in that period. Cluster6 (complementary medicine) also features several research hotspots in recent years, some of the main hotspots include depression, non-infectious diseases, chronic pain, multidisciplinary care planning, and mind–body practices such as meditation and yoga.

## 4. Discussion

We used various bibliometric tools to provide a comprehensive bibliographic review of IC from the Web of Science (WOS) from the period 2010–2020. Through approaches such as keyword frequency analysis, co-authorship analysis, reference co-citation network analysis, and highly cited articles analysis, we demonstrated the focus, prospects, and challenges of IC research.

### 4.1. Trends of interest and disciplinary distribution in IC research

Over the past 10 years, although the IC field has received increasing attention, the degree of attention has been gradually decreasing every year (Figure 1). This could be attributed to various factors, and one possible reason is that it has become more widespread, thus the initial novelty is fading and people are shifting their attention to other medical issues and priorities. While the attention given to IC may be decreasing, it is important to recognize its value and potential in improving healthcare outcomes. In terms of disciplinary area distribution (Figure 2), IC is widely distributed in multiple fields, such as health care sciences services, public environmental occupational health, health policy services, general and internal medicine, psychiatry, oncology, nursing, pharmacy and pediatrics, reflecting that the breadth and depth of IC applications are constantly expanding. The wide distribution of IC in various fields presents a unique opportunity for collaboration and knowledge exchange. For instance, healthcare professionals from different fields can share their insights and experiences in implementing IC in their respective fields, thus enhancing the development and delivery of IC services.

### 4.2. Dominance of developed countries in IC research and importance of tailoring IC strategies to specific settings

In terms of the number of publications and the BC value (Figure 3), the top three countries were the United States, the United Kingdom and Australia. Combined with Table 1, the proportion of developed countries’ publications is much higher than that of developing countries. Many high-income countries in Europe and America have adopted IC to address the health needs of aging populations and rising rates of chronic and multiple morbidities (Point, 2011; Kringos and Klazinga, 2014). For instance, IC has been available in the United States for 76 years, since Kaiser Permanente began offering the first IC insurance plan in 1945 (Craig et al., 1999; Laugesen and France, 2014). The English National Health Service has experimented with the integration of care since the 1990s and established 16 IC pilot programs with a range of objectives in 2008. To some extent, there is no one model of IC strategy that can fit all countries (Averill et al., 2010; Maruthappu et al., 2015), the design of specific measures should take into account the local culture, socioeconomic development, governance, and service recipients (Atun et al., 2010; Stadnick et al., 2019). However, even if different countries have different paths to achieve IC, there is a need to develop a uniform framework of principles. WHO has developed a common set of principles of IC that are comprehensive, equitable, sustainable, holistic, preventive, empowering, respectful, collaborative, co-produced, endowed with rights and responsibilities, governed through shared accountability, evidence-informed, led by whole-systems thinking and Ethical (World Health Organization, 2015). Furthermore, value-based healthcare has become an international consensus (Daniels et al., 2022; de Vasconcelos et al., 2022; Kokko, 2022). While WHO provides a thorough and widely-accepted IC framework, but there’s potential for further refinement, context-specific adaptation, and inclusion of new concepts as healthcare evolves. The principles’ effectiveness hinges on their practical implementation in real-world healthcare scenarios. Thus, countries should collaborate to enhance the value objectives, principles, and key elements of IC, and explore paths of integration tailored to each nation’s characteristics within the theoretical framework.

### 4.3. Co-authorship networks and the need for improved collaboration in IC research

Bibliometrix R-tool was used to analyze the top 20 most productive authors (Table 2), Ucinet was used to manage the co-author matrix. Ben-Arye E had the most publications but a BC of 0 and was not placed in the control position in the network information; the author formed a triangular cooperation network with Schiff E and Jacobsen SJ. On the other hand, although Quesenberry CP only published 31 articles, he collaborated with several authors to form a large cooperative network. In addition, he had the largest BC and was at the center of the network, meaning that he acts as a bridge for information in the network. The analysis reveals that effective cooperation among authors is lacking, despite the formation of several scholar groups. This finding indicates a need for greater collaboration within the academic community, by promoting more effective collaboration, including theoretical exchanges and practical sharing, researchers can pool their expertise and resources to accelerate advancements in the field.

### 4.4. Interdisciplinary collaboration and trends in IC research

From BibExcel statistics, 93 high frequency keywords were extracted (Table 3). In addition to “integrated care” and “integrated health care,” the hot keywords included “primary care,” “mental health,” “complementary and alternative medicine,” “palliative care,” and “self-management.” By using the knowledge mapping tool VOSviewer to visualize keyword co-occurrence analysis (Figure 6), four further hotspot clusters were generated (Table 4). These results indicate that IC is not limited to the clinical field, but has broken down disciplinary barriers and encourages interdisciplinary cooperation, thus health systems should provide health services that integrate prevention, diagnosis, treatment, rehabilitation, and palliative care to cover the health needs of the region’s residents. In addition, to meet the needs and preferences of the population, individuals should be seen as active participants in health management and take responsibility for their own health (Rittenhouse and Shortell, 2009; Singer et al., 2011).

Science knowledge mapping was used to analyze reference co-citation, highly cited articles and timelines and detect and research historical evolution and trends. With the support of CiteSpace, 210,081 references were analyzed. According to document co-citation analysis, seven clusters were generated (Figure 6), namely, Cluster 0 (qualitative study), Cluster 1 (serious mental illness), Cluster 2 (health systems integration), Cluster 3 (integrative medicine), Cluster 4 (patient-centered medical home), Cluster 5 (chronic pain) and Cluster 6 (complementary medicine). We observed that the prominence of Cluster 0 (qualitative study) as the main cluster in keyword analysis could be due to its vital role in integrated healthcare research, where it is often used to investigate patient experiences, healthcare behaviors, and healthcare decision-making. Furthermore, we noted that the earliest research clusters are Cluster 3 (integrative medicine) and Cluster 4 (patient-centered medical home), which are related to the history of integrated healthcare development, from advocacy IHCD to encourage health systems to embrace PCIC approach to organizing health services. Notably, “complementary medicine” has become a hotspot in recent years, especially with the increasing prevalence of cancer, chronic diseases and mental illnesses; integration of complementary and western medicine can improve the quality of life of patients (Mongiovi et al., 2016; West, 2018; Saeed et al., 2019; Hübner, 2020). In addition, traditional Chinese medicine plays a positive role in the treatment of COVID-19 (Ren, 2020; Zhang et al., 2020). Thus, complementary medicine will continue to be a focus in the future, however, it should be noted that the use of CAMs in clinical practice needs to assess the effectiveness, and safety of CAM (Paoloni et al., 2022).

### 4.5. Key contributors and publications in the IC field

Among the top 10 frequently cited articles, four were concerned with integrated or integrative care, and the other articles were about pediatrics, collaborative care, complementary and alternative medicine, highlighting the interdisciplinary nature of IC. These highly cited articles were contributed by influential authors such as Valentijn PP, Koebnick C, Berwick DM, Barnett K and Woltmann E, who have made significant contributions to the development and advancement of the field, enhancing our knowledge and understanding of IC. The sources of articles with more citations and higher influential factors included the Lancet, the New England Journal of Medicine, the International Journal of IC and the American Journal of Psychiatry. High-quality periodicals have a positive effect on the development of the subject. These journals provide a platform for disseminating research findings and facilitating collaboration among researchers and healthcare professionals, contributing positively to the development of the field.

### 4.6. Challenges and solutions in advancing IC

The advancement of IC faces different challenges, especially in complex and changing external environments. Firstly, theory and practice are not in sync. Person-centered care is often defined as the core of IC, so people-centered care needs people-centered research, IC strategies should be based on co-creation with patients or citizens in ways that involve, engage and empower them, but a chasm remains between theory and practice (van der Vlegel-Brouwer et al., 2020). In addition, inter-organizational collaboration is the key to delivering IC. However, many barriers related to administration and regulation, resources and funding hinder the collaboration (Andersson et al., 2011; Koebnick et al., 2012; Boothroyd et al., 2015; Auschra, 2018; Simpson et al., 2023), Therefore, relevant authorities should take action to overcome such barriers. For example, China has proposed the “County Medical Alliance,” which integrates the medical service network at the county, township and village levels in six areas: administration, personnel, finance, services, assessment and supply of medicine and equipment to provide a full, continuous and coordinated service for service recipients. Countries or regions with health service systems similar to those of China can learn from the “County Medical Alliance” model to promote the development of IC. Notably, financial incentives are potentially powerful tools to stimulate IC (Averill et al., 2010; Tsiachristas, 2016), but separate payment mechanism can block effective integration (Struckmann et al., 2017), moreover, each payment method has a certain negative incentive effect, so mixed payment should be adopted to support more effective and efficient IC system (Berenson and Rice, 2015; Stokes et al., 2018). Furthermore, silos of data collection on a cross-institutional level have created a fragmentation of electronic medical records in many countries, potentially hindering continuity of IC and resulting in clinical, and administrative inefficiencies (Bradley et al., 2017; Meinert et al., 2019; Eh et al., 2020). Governments should fully recognize the great value of medical data, strengthen information systems and promote legal and compliant medical data sharing.

## 5. Study limitations

The present study had several limitations that need to be mentioned. Firstly, we only focused on articles that have been published in WOSCC and excluded non-English articles or neglected other forms of publication (e.g., books, web pages, and policy documents), which might cause the data obtained to be not comprehensive enough. Additionally, although bibliometrics could provide a valuable mix of information to reflect the current status and research hotspots of the IC domain, it cannot reveal the overall situation in the field, especially when other methods, such as the Latent Dirichlet Allocation model, which can be used for qualitative examination, are ignored. Therefore, Future research could expand our study’s findings by including non-English publications and various formats, using additional bibliometric tools like the Latent Dirichlet Allocation model, focusing on the application of identified IC principles and models in diverse healthcare settings, and integrating qualitative research methods for a more comprehensive understanding of the research landscape, emerging themes, and trends.

## 6. Conclusion

In conclusion, the global field of IC has expanded and achieved remarkable results in the last 10 years. Developed countries pay more attention to the development of IC than do developing countries, and minimal cooperation occurs among authors and institutions. In addition, results indicated four hotspot clusters, namely, IC, depression, integrative medicine and primary health care. Combined with cluster timeline visualization, complementary medicine has become a hotspot in recent years and will continue to be a focus. Furthermore, there are still many challenges and barriers to IC to achieve person-centered care, these include, but are not limited to resources fragmentation, efficient collaboration difficulties between different levels of health institutions, insufficient financial incentives, inadequate self-management in health, and poor information sharing. So international collaboration should be further strengthened to promote the development of integrated healthcare with value co-creation and model innovation. These findings will help scholars better identify new perspectives for future research.

## Author contributions

DG, HL, DS, and YC designed the study. DG, CZ, and XC performed the data analyses. DG, CZ, GG, and XC wrote the initial draft of the paper. All authors contributed to the article and approved the submitted version.

## Funding

This work was supported by the National Natural Science Foundation of China (Support batch number: 71974066) and “Double First-class Construction Project of Liberal Arts in Huazhong University of Science and Technology” (Think Tank of Rural Health Service Policy and Management).

## Conflict of interest

The authors declare that the research was conducted in the absence of any commercial or financial relationships that could be construed as a potential conflict of interest.

## Publisher’s note

All claims expressed in this article are solely those of the authors and do not necessarily represent those of their affiliated organizations, or those of the publisher, the editors and the reviewers. Any product that may be evaluated in this article, or claim that may be made by its manufacturer, is not guaranteed or endorsed by the publisher.
